# Developing a framework for Swiss outpatient pediatric research network

**DOI:** 10.3389/fped.2025.1561070

**Published:** 2025-06-24

**Authors:** Chantal Kuske, Salvatore Maione, Emiliano Soldini, Michael von Rhein

**Affiliations:** ^1^ Child Development Center, University Children's Hospital Zurich, University of Zurich, Zurich, Switzerland; ^2^ Department of Business Economics, Health and Social Care, University of Applied Sciences and Arts of Southern Switzerland, Manno, Switzerland

**Keywords:** pediatrics—children, participatory research, research network, healthcare service, outpatient care, primary care

## Abstract

**Aims:**

In Switzerland, availability of pediatric outpatient data for research is limited. Pilot projects showed the benefits of collaborative networks for research, but there is no common understanding on how to best organize, govern, operate and fund these. This project aimed at developing a framework for the establishment of a nationwide collaborative outpatient pediatric research network.

**Methods:**

Following a qualitative approach, we conducted individual interviews, a workshop and a focus group with pediatricians and other healthcare stakeholders to discuss various aspects related to the development of the research network, including motivations for participation, retention strategies, perceived barriers, expected challenges, and previous experiences.

**Results:**

Participants were interested and willing to join such a network and gave valuable inputs, but also emphasized important challenges, particularly time constraints/limited resources, data management/IT infrastructure and funding. These insights allowed developing the outline of a three-step iterative implementation plan.

**Conclusions:**

The project emphasized key elements to consider for the development of a Swiss outpatient pediatric research network sustainable in the long term, that would mark a pivotal advancement for pediatric healthcare research.

## Introduction

According to the Population and Households Statistics (STATPOP) of the Federal Statistical Office (FSO), at the end of 2023 Switzerland had a population of approximately 9 million individuals with a median age of 42.7 years and a total fertility rate of 1.4 children per woman (1). The country boasts a significant pediatric healthcare workforce, with more than 1,300 pediatricians (61%) operating in private practices (2), caring for 1.3 million children and adolescents under the age of 15 years (i.e., 14% of the total population). Yet, despite this substantial presence, the paucity of data from the pediatric outpatient care sector for research purposes is striking.

Switzerland's pediatric research is primarily coordinated through SwissPedNet, a national network linking pediatric hospitals and academic institutions to support high-quality, multicenter clinical trials. While SwissPedNet—aligned with the Swiss Clinical Trial Organization and collaborating with European networks like conect4children (c4c)—covers various subspecialties, it currently lacks a focus on pediatric primary care research, as do similar networks in Germany, the UK, and the US. In 2020, the Federal Office of Public Health highlighted the imperative to bolster health services research in Switzerland (3). An interview study by Ormond et al. (4) further highlights the legal, ethical and logistical challenges of health data sharing in Switzerland, suggesting that uncertainties around data ownership, anonymization and privacy laws are significant barriers to health data sharing. However, with few exceptions, initiatives launched thus far have featured limited inclusion of pediatric data from the outpatient sector, despite many organizations from the academic and primary care sectors, such as the Kollegium für Hausarztmedizin (KHM), SwissPedNet, Kinderärzte Schweiz (KIS), Swiss Pediatrics, and University Children`s Hospitals, recognize the importance of research in the outpatient care sector (5, 6). To date, the predominant focus of pediatric studies remains within the hospital environment, resulting in a notable absence of valuable perspective and data originating from outpatient care settings and representing a significant gap in healthcare research, particularly in the context of children and adolescents.

Considering these challenges, the establishment of a nationwide research network of collaborative pediatric care practices is called for. Such an initiative could address research questions affecting the daily activities of primary care pediatricians. Moreover, it could possess the potential to collect structural data from the pediatric outpatient sector, a vital asset from the vantage point of public health. By doing so, pediatric outpatient research could play a crucial role in enhancing child health outcomes and optimizing healthcare delivery (7). Recent studies by Geary et al. (8), Rajamani and Iyer (9) and Arnold et al. (10) underscore the importance of well-structured networks and standardized data collection in improving healthcare quality and coordination. These findings highlight the need for a comprehensive pediatric research network to address gaps in outpatient care. Embarking on this journey, two pioneering pilot projects have taken root in the Cantons of Zurich and Ticino. In Zurich, over 40 pediatric practices, united under the “SentiPED” network, collected longitudinal data on SARS-CoV-2 testing and clinical insights into afflicted pediatric patients during the pandemic (11). This ambitious effort was fortified by the endorsement of Kinderärzte Schweiz, encompassing structural data from participating practices. Likewise, Ticino witnessed the successful implementation of a parallel data collection initiative, “Sentinella Pediatrica”, orchestrated by the Associazione Pediatri della Svizzera Italiana (APSI), which thoughtfully incorporated documentation on the burgeoning concern of long COVID (12). The significance of these localized endeavors is undeniable, serving as a propitious foundation upon which to merge the efforts of collaborative networks of primary care pediatricians. The aspiration to expand these networks to encompass a nationwide scope beckons, but this pursuit mandates careful preparation, starting with the harmonization of governance structures, funding strategies, decision-making processes, and collaborative modes. This crucial groundwork would pave the way for the organic evolution of a dynamic and impactful nationwide research network for primary care pediatricians. Swiss School of Public Health (SSPH+) has also recognized this need and enabled the development of a corresponding white paper through project funding.

The main aim of this paper was to describe the basic framework for establishing a nationwide research network of private practice pediatricians and set the stage for future research efforts, addressing the key aspects related to the network's development.

## Materials and methods

Our core working group consisted of a convenience sample of 12 experts in the field of pediatric health care research—6 women and 6 men located in the regions of Basel, Bern and Zürich—with tight connections to primary care pediatricians, pediatric hospitals, professional associations and academia. More specifically, the working group counted 7 pediatricians, 1 pediatric cardiologist, 1 physician specialized in rare diseases, 2 clinical trial coordinators and 1 representative of the Federal Office of Public Health (FOPH). Four of these were also active in academia as professors or researchers, while 2 were representatives of professional associations/organizations.

We relied on a three-phases qualitative research approach, with initial individual meetings that were followed by an in-person workshop and a final online focus-group discussion. Key stakeholders from across Switzerland were identified based on formal and informal networks invited to participate in the abovementioned activities. After searching the literature (both scientific and grey publications) for examples of existing research networks in primary care and respective initiatives in Switzerland, we conducted, between March and August 2023, a series of individual online meetings with all members of the core working group. In these individual online meetings, we presented background and aims to our target audience (primary care pediatricians), and asked for their insights, feedback, and interest in potential collaborations. The topics discussed covered possible motivations for joining a national pediatric research network, reasons for conducting participatory research in pediatric primary care, challenges to expect, and experiences with previous study participation or involvement in regional or national research projects. These interactions allowed for in-depth exploration of their perspectives. The issues and questions raised in the interviews were then grouped, edited, and used as basis for the discussions at an in-person workshop held at the SSPH+ Faculty Meeting in Basel on June 20, 2023. After presenting the overall aims and results from the interviews to the audience, composed of several pediatricians, public health experts and other healthcare stakeholders (approximately 20 persons), the participants were divided into five groups to discuss open questions regarding the network implementation, namely professional profiles to be included, strategies for participants' recruitment and retention, communication and dissemination of research findings, long-term financing, and integration with other existing data networks. As a last step, 5 pediatricians of the working group participated in an online focus group discussion on November 6, 2023. An initial presentation of the overall network concept and of the aims of the white paper was followed by a semi-structured discussion aimed at exploring motivations, perceived challenges and facilitators related to their involvement in the research network.

## Results

Individual meetings involved a diverse group of eight professionals, ranging from residents and general practitioners to clinical trials coordinators. Sharing initial insights with similar projects, all participants expressed a strong interest in the initiative and highlighted the urgent need to establish clear rules and responsibilities for a national outpatient pediatric research network, including the provision of the necessary infrastructure. Inspired by the existing structures and regulations of hospital-based research projects, one of the most promising approaches identified was the streamlining of research processes adapted to our ambulatory research network. They also expressed their personal motivations in favor of an outpatient pediatric research network, which were the gathering of representative data for insurance and government purposes, the improvement of the research quality and daily practice, and the enhancement of representation. However, they also identified barriers such as limited time in practice, challenges with data sharing, and obtaining ethical approval. In addition, these initial discussions served to disseminate information about the project in professional circles (Table 1).

**Table 1:** Topics discussed and points raised by the participants at all three activities.

Format	Topics addressed	Input from participants (bullet points)
Individual interactions between March and August 2023	Sharing the project idea of the national outpatient pediatric research network and asked for feedback and interest	- Strong interest in the initiative - Urgent need for clear rules and responsibilities - Necessity for infrastructure provision - Dissemination of information within professional circles, for workshops - Representative data for insurance and government purposes - Improvement of the research quality and daily practice
	Experience with Similar Projects	- SwissPEDNet & Kinderärzte Schweiz - Inspiration from hospital-based research structures and regulations of University Children`s Hospitals
	Barriers	- Limited time in practice - Challenges with data sharing - Difficulties in obtaining ethical approval
Workshop SSPH+ faculty meeting Basel June 20, 2023	Reasons for joining and facilitators	- Improvement of practice quality - Strengthen negotiating positions with insurance companies
	Key professional for the network	- Pediatric outpatient clinicians, data managers, IT experts, communications specialists, policy experts, and health insurance professionals
	Challenges and resolution approaches	- Resource constraints among outpatient pediatricians - Strategies include understanding motivations, minimizing effort, and providing various incentives - Keeping the effort small for pediatricians and providing incentives
	Strategies for private practice recruitment and retention	- Tailoring pilot projects to pediatric hot topics - Offering both monetary and non-monetary incentives to motivate participation - Include all relevant stakeholders - Shared database with standardized coding
	Integration/harmonization with existing data networks strategies	- Employing standardized coding for data integration and harmonization - Promoting a collaborative approach to benefit from a shared database - Referencing existing networks like Swiss ORCHID, the FIRE network, EPR, and FMH Swiss Medical Association as infrastructure examples or future collaboration candidates
	Focus on initiating high-interest projects for outpatient pediatricians	- Identifying and addressing high-interest topics through tailored pilot projects - Leveraging the network to facilitate research that responds to the specific needs and interests of outpatient pediatricians - Research questions should come from pediatricians
	Existing network and registers	- Swiss ORCHID, the FIRE network, Electronic Patient Record (EPR), and FMH Swiss Medical Association
	Communication strategies	- Different channel such as: website, newsletter, social media, fact sheets, blogs, media, short videos
Focus group discussion Online November 6, 2023	Joining reasons and facilitators	- Collecting data - Involvement in research projects - Support for political and insurance decisions - Time and resource constraints
	Identifying challenges	- Supporting data collection and statistics - Data anonymization and ethical approval
	Pilot project ideas for the research network	- New care models with fewer pediatricians and more children - AI usage - Standardized checklist

The workshop at the SSPH+ faculty meeting again highlighted the need and interest in establishing a national pediatric outpatient research network. Participants furthermore described key professional profiles, which they recommended to include in the network (i.e., pediatric outpatient clinicians, data managers, IT experts, communications specialists, policy experts and health insurance professionals). Suggestions for strategies to recruit and retain private practices included understanding pediatricians' motivations, keeping their effort small, and providing incentives. For instance, pilot projects could motivate pediatricians to participate in the network; these should be mainly tailored to address the principal interests and needs of the network members (e.g., treatment of rare diseases in the outpatient setting, adoption of AI tools to enhance patients' management). In addition, other non-monetary incentives (e.g., assistance with data collection and analysis, support in pursuing grant applications or obtaining ethical approval for studies) were discussed as possible facilitators since outpatient pediatricians often face resource constraints limiting their ability to participate in or carry out research projects. The prospect of using data to improve practice quality or strengthen negotiating positions with insurance companies was also discussed as other potential motivators. Also, the affiliation to a research network could be regarded as a proof of quality. As another topic, data integration and harmonization strategies (using standardized coding) were discussed to promote a collaborative approach in which multiple stakeholders could benefit from the access and use of a shared database resulting from the aggregation of single outpatient pediatrician data. Participants saw substantial benefits in disposing of more data to address research questions and increase quality standards. During the workshop, several existing networks and registers (e.g., Swiss ORCHID or the FIRE network, see Table 1) were referred to as both examples of possible infrastructures and candidates for future collaborations. Also, the importance of different communication and dissemination channels, such as websites, newsletters or social media, was highlighted (Table 1).

The online focus group discussion confirmed all the above-mentioned aspects (i.e., strong interest in collecting data, becoming involved in research projects, time constraints for pediatricians, and need for support in data collection and statistics). Additionally, participants described concerns about anonymity, raised the idea to use existing standard clinical data for research purposes, and highlighted that their contributions in research projects should be acknowledged. Project aims should be targeted to improve political and insurance support for patients, for example in the field of rare diseases. As potential pilot projects, participants saw great potential in topics like “new care models with fewer pediatricians and more children” and “AI use and standardized checklists” (Table 1). Both project ideas could basically target a fundamental current issue in the outpatient pediatric setting, namely the scarcity of resources in the daily practice. In this sense, the projects could explore the feasibility and effectiveness of the introduction of new organizational models, professional figures (e.g., advanced practice nurse) and technological tools—as well as the systematic adoption of standardized validated checklists and national and international guidelines frameworks—for patient assessment and management.

The exchange with the stakeholders in the different formats highlighted three main challenges, namely time constraints/limited resources in the context of (1) participant engagement, (2) data management/IT infrastructure, and (3) funding.

Regarding participant engagement, all agreed that pediatricians must be unburdened from additional work (or, at least, supported). In this sense, retrospective studies or specific data collections related to projects promoted by network members (whose implementation modalities would be defined within the network) should be preferred, at least at the beginning, to minimize the additional workload. Participation in interventional clinical studies could be envisaged only at a later stage, with the network robustly established and already integrated with other existing clinical networks. Customized recruitment and retention strategies were also discussed. Personalized incentives were highlighted as being fundamental since participation in the network could be related to several reasons. For example, some stakeholders would be more interested in writing scientific publications while others in discounted/free lifelong education programs or financial incentives to provide data.

All agreed that data management must be organized in accordance with the Federal Act on Research involving Human Beings (HRA) but also should allow open accessibility (as far as possible). Concerning IT infrastructure, participants agreed that the network would probably be composed of members with different data management systems, making it difficult to think of immediately developing an automated data collection system. A centralized data registry (e.g., based on REDCap) would be more feasible, at least in the initial setup. On the long run, a link to initiatives for data sharing and interfaces to existing data collections were envisioned.

**Figure 1:**
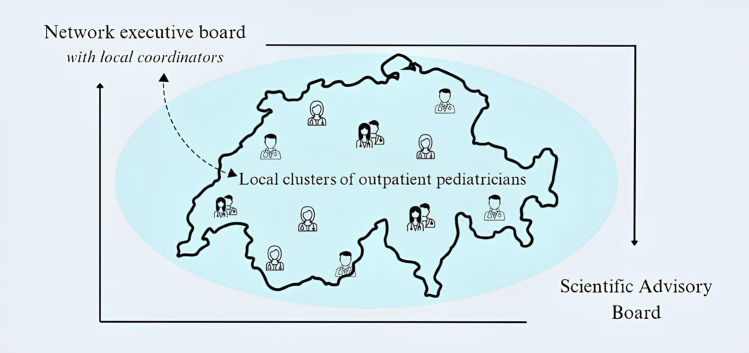
Proposal of the network infrastructure.

Regarding funding strategy, participants agreed on submitting specific research projects to competitive funds (e.g., SNSF) at the beginning. Subsequently, with the growth of the network, management-administrative structure would be needed to plan and support research programs. At this stage, fundraising policies, including public and private partners other than industry, would be essential to guarantee a sufficient continuity of funding (e.g., FOPH, Childhood Cancer Research Foundation Switzerland). Industry funding to foster outpatient participation in clinical trials could be considered only after a certain degree of maturity and integration of the network with other realities have been reached. Based on these premises and our initial literature research, we developed a preliminary outline of the network structure, coordinated by a central network executive board which is advised by a scientific advisory board and communicates to smaller network clusters via local coordinators as described in Figure 1.

The scientific advisory board would provide advice on the general strategic directions of the research network. The members of the strategic board would come from different sectors (academic and clinical, public and private companies) to provide a broad expertise. For example, it would be beneficial to also include in this group representatives of hospital pediatrics or patients/parents in addition to outpatient pediatricians. The executive board would decide on the overall strategy, infrastructure, funding, and other fundamental questions, as well as on aims and design of specific research projects, and would oversee the correct execution of the network's research activities. It would have the overall responsibility for the network project, including the legal responsibility for the financial, contractual, initiation and management aspects. The responsibilities would cover the contracting of funders to pool resources and the agreements with the research network to guarantee smooth, scientifically sound and sustainable implementation of the research infrastructure. Besides pediatric expertise, this board should incorporate competencies in aspects of law and compliance, IT, finances, organization and management. Local coordinators should be the executive board members that would also be in charge of supervising the activities conducted in the local clusters. The local clusters would represent the point of contact and aggregation of the pediatricians located in the area. The clusters should be responsible for implementing the research activities proposed by the executive board and to promote the network activities in the local communities (e.g., regional pediatric associations, dissemination promotion of life-long learning and research results) and the regional recruitment campaigns. Beyond research projects promoted by the executive and strategy boards, additional projects and/or activities could also be proposed by single members/clusters of the network within a participative approach. A concrete implementation plan was outlined as described in Table 2.

**Table 2:** Implementation plan of the Swiss national research network group.

Stage 1: Pilot project	1. Selection of a research question, and adequate methods/design 2. Applications for funding 3. Kick off with a reduced version of the administrative structure of the network (temporary scientific and executive boards, agreement documents, IT infrastructure, etc.) 4. Recruitment of a test cluster of pediatricians (ideally, Italian-, French- and German-speaking regions of Switzerland) to participate in the pilot project 5. Pilot project data collection and analysis 6. Evaluation of the preliminary network version, stakeholder engagement strategies, data management policies, IT infrastructure approaches and fundraising 7. Dissemination of the results, including the creation of a lifelong education project
Stage 2: Development & consolida the networktion of	1. Establishment of the definitive administrative structure of the network 2. Development of a sustainable research program including funding 3. Further program-related phases of members’ recruitment to seek the highest possible representativity at the Swiss level (e.g., canton/region, urban vs. rural, etc.) 4. Carrying out further individual projects 5. Dissemination of the results, including the strengthening and development of the lifelong education project 6. Revision and adaptation of the stakeholder engagement strategies, data management policies, IT infrastructure approaches and fundraising plan for the next implementation phase
Stage 3: Future steps	1. Evaluation of the necessity of separating the executive and funding board into two separate entities (one dedicated to activities’ organization and the other one to funding management) according to the expected level of funds to administrate 2. Points (2–5) of Stage 2

## Discussion

Given the need to collect research data from the field of pediatric primary care and the limited availability of outpatient pediatric data for research purposes, we aimed at describing a basic framework for establishing a nationwide research network of pediatricians in private practice in Switzerland, based on various sources of information. After conducting an initial review on existing data collection or research networks in primary care, we invited stakeholders with a broad spectrum of backgrounds to discuss the key aspects of such a network. After that, we integrated the insights from these sounding boards into the existing structures and examples and adapted them according to the specifics in pediatric primary care in Switzerland, leading to the outline of a three-step implementation plan. Participants were willing to join such a collaborative network and gave valuable input but also emphasized important challenges. We believed that the conceptual framework presented, and the establishment of the respective infrastructure could help to promote the involvement of primary care practitioners in pediatric research in Switzerland. Time constraints and limited resources were central topics, brought up by our participants. Delivering high-quality patient care within limited time and resources leaves minimal room for the additional commitments that robust research demands. Pediatricians practicing outside hospital settings described resource deficits and expressed the need for support, e.g., by study nurses. This scarcity significantly hinders various research-related tasks, such as grant applications or seeking ethical approvals. Moreover, the challenge of time (and funds) allocation for research is compounded by the limited patient engagement already inherent in non-hospital settings. In addition to these constraints, there is a further major challenge related to the absence of a universal electronic data repository useful to easily share research data. Such data sharing activities would imply the development of detailed data management policies, in terms of ownership, access, usage and sharing. To overcome these challenges, establishing a robust research network in Switzerland would be essential. Furthermore, the suggested framework structure could be used as an example for initiatives to set up comparable frameworks elsewhere. Pediatric outpatient research could play a crucial role in enhancing child health outcomes and optimizing healthcare delivery, e.g., by understanding real-world treatment effectiveness, patient adherence, and safety profiles. By focusing research efforts on these settings, clinicians could gather data that more accurately reflects everyday clinical practice, leading to improved therapeutic strategies and patient care. This approach not only would benefit individual patient care but would also contribute to public health by informing policy decisions and resource allocation, as argued by Laviolle et al. (9). A recently published systematic review highlighted the evidence for various benefits of research networks in healthcare, regarding the treatment, referral, and best-practice care of patients (10). The success of the SwissPedHealth project “Preparing PERsonalizEd PediatRic PrimaRy care (PREPP)” stood as a compelling exemplar within this context (13). The aim of PREPP was to integrate routine primary care data with the national SPHN infrastructure. This initiative significantly enhanced the quality of local health data by merging hospital and primary care data, offering direct benefits to patients. It facilitated rapid access to critical patient information for emergency departments and allowed participating in primary care sites to share and receive patient information in real time, demonstrating the practical benefits achievable through effectively integrating health data networks. Furthermore, the ongoing project initiated by the Federal Office of Public Health (FOPH), titled “Child and Adolescent Health—Minimal Set of Indicators for Switzerland,” advocates for the importance of our project (14). This initiative, which directly addresses the needs of pediatricians in private practice, highlights the crucial role of collaborative efforts. It serves as another key example of how structured projects (e.g., Pediatric Research in Office Settings, PROS) (15), can significantly contribute to complete the pediatric healthcare landscape. Finally, the FIRE project (16), a comprehensive database aggregating routine of primary care data across Switzerland, also justifies our initiative since the pediatric sector currently lacks such a unified data structure for collaborative research efforts. These examples underscore the profound impact that a well-structured pediatric research network can have and the urgent need for coherent data management and data sharing strategies. Addressing the complexity of these challenges requires an iterative and participatory process, involving the continuous development, discussion and revision of various aspects of the network.

The above-mentioned projects are intended to facilitate the integration and use of data for research and activities for the improvement of quality of care. Although they facilitate the implementation of projects, they do not have the main objective of actively promoting research and involving various stakeholders. Our project aimed to fill this gap by considering, in addition to the collection and harmonization of data, the design, financing and execution of research programs as their main purpose. The establishment of such a network could be therefore essential to promote the development of research activities in the outpatient sector and the active involvement of the various field stakeholders in the long term to establish a shared philosophy of research. Integration with other initiatives at national and international levels could be considered and implemented (with modalities to be defined) once the network has been reached a well-defined and sustainable structure, so that integration activities could bring mutual benefits to all partners involved, especially for early intervention and chronic care management (7).

While this paper outlined the strategic importance and anticipated benefits of a nationwide pediatric research network in Switzerland, it may not fully address the complexities and potential barriers in practical implementation. In this sense, further research efforts particularly in the areas of advanced data analytics, collaborative platforms for healthcare professionals, and funding for ongoing research would be required. Moreover, given that the perspectives highlighted were limited to professionals active in the Swiss German area, further research must involve other stakeholders of the French and Italian regions to provide a more representative assessment of the situation.

## Conclusions

In conclusion, the establishment of a national research network for primary care pediatricians in Switzerland would mark a pivotal advancement in addressing the existing gaps in pediatric healthcare research. The collective drive and enthusiasm evidenced in our workshops and meetings underscored a deep-rooted commitment to enhancing pediatric outpatient care. Confronted with challenges such as intricate data collection processes, the absence of a comprehensive electronic database, and resource constraints, the need for this network has become increasingly evident.

This project could have a significant impact pediatric healthcare outcomes. By fostering collaboration, aligning research with the practical realities faced by practitioners, and establishing a framework for streamlined data sharing, we aimed to elevate the quality of pediatric care. This initiative has been particularly focused on addressing real-world clinical queries and establishing a rich and impactful pediatric research landscape in Switzerland.

The future of this project would be shaped by several key objectives: promoting collaboration among pediatricians and outpatient care stakeholders, optimizing the use of existing resources, establishing a platform for continuous education in pediatric research, and supporting the practical implementation of research within ambulatory care settings. Additionally, by strengthening the ties between inpatient and outpatient pediatric research, we would aim to create a more integrated and effective research landscape, which would be fundamental to improving health outcomes for children.

Ultimately, the vision of this project was to forge a synergistic and efficient network, not just to advance pediatric research but to ensure these advancements could be translated into tangible benefits for pediatric healthcare. This network would stand as a testament to our commitment to bridging the gap between research and practice, ensuring that the future of pediatric care would be shaped by evidence-based, collaborative, and innovative approaches.

## Data Availability

The raw data supporting the conclusions of this article will be made available by the authors upon request.
